# Questioning the kidney-protective effect of tolvaptan: a biologically plausible hypothesis to revisit the current narrative

**DOI:** 10.12688/openreseurope.20428.1

**Published:** 2025-06-04

**Authors:** Dion Groothof, Stephan J.L. Bakker

**Affiliations:** 1 Internal Medicine, University Medical Center Groningen, Groningen, Groningen, 9713 GZ, The Netherlands

**Keywords:** ADPKD, Tolvaptan, Creatinine, Cystatin C, Muscle mass, Kidney function, Confounding

## Abstract

Tolvaptan is the only approved therapy for slowing disease progression in autosomal dominant polycystic kidney disease (ADPKD). Its use necessitates a careful balance between its kidney-protective effects and the burdensome aquaretic effects, which significantly impact quality of life. Real-world data highlight that a substantial proportion of patients decline therapy due to these side effects and long-term adherence remains a major challenge. For patients unable to tolerate or unwilling to initiate treatment, the psychological burden of guilt and self-blame for potentially forgoing kidney protection is likely profound.

In this perspective article, we critically examine the kidney-protective claims of tolvaptan. We hypothesize that use of tolvaptan can induce loss of muscle mass and that a reevaluation of data from the TEMPO3:4 trial using cystatin C measurements—an alternative endogenous filtration marker next to creatinine that is unaffected by changes in muscle mass—may reveal no significant benefit of tolvaptan in slowing kidney function decline compared with placebo. If confirmed, these findings would prompt a reassessment of tolvaptan’s role in ADPKD management. More importantly, it could alleviate the emotional distress borne by some patients and families unable to pursue therapy. This underscores the urgent need for rigorous validation of tolvaptan’s efficacy, ensuring therapeutic decisions are guided by robust evidence and grounded in both clinical and humanistic considerations.

## Disclaimer

The views expressed in this article are those of the author(s). Publication in Open Research Europe does not imply endorsement of the European Commission.

**Figure 1.**
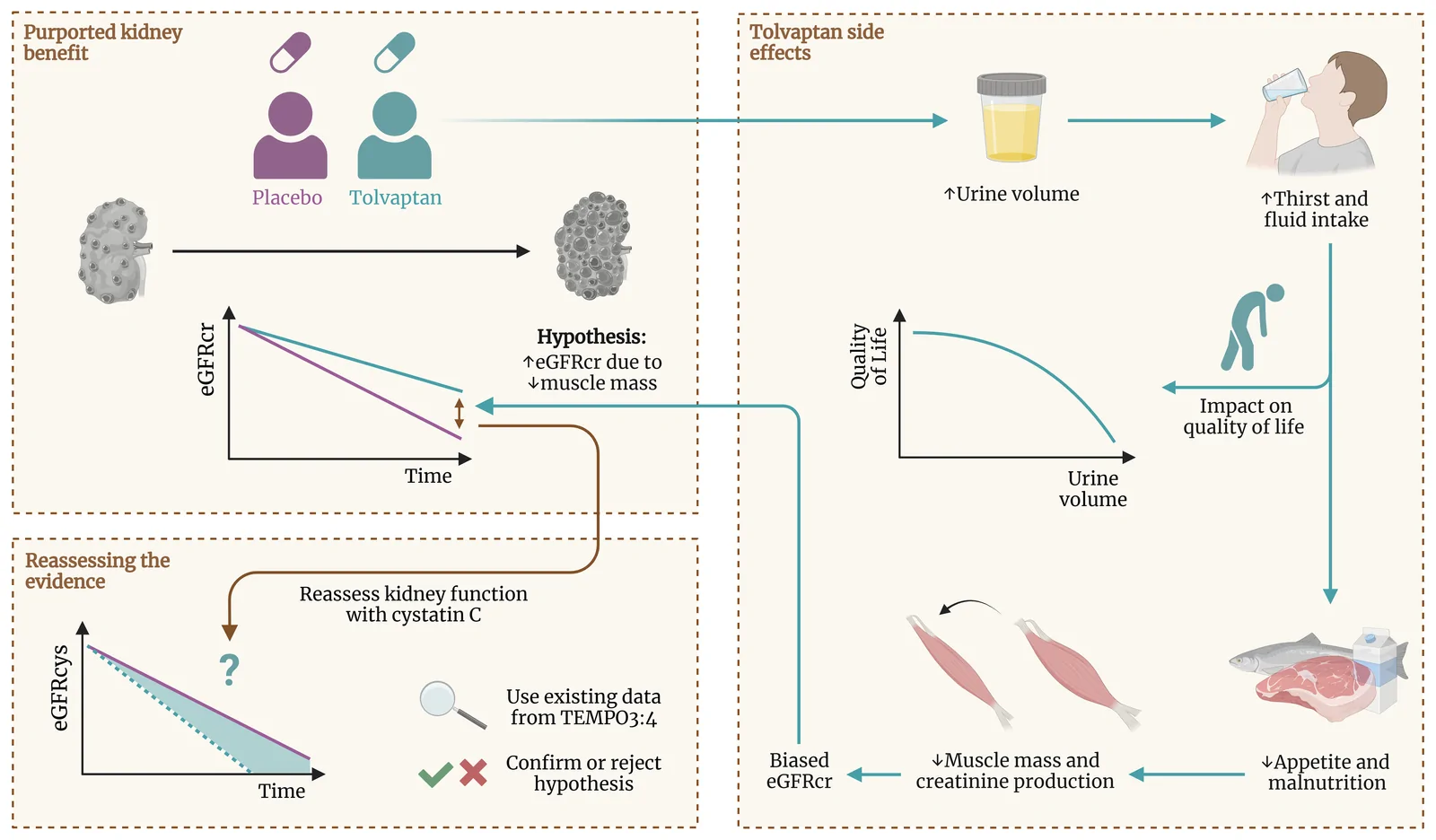
Hypothesized mechanism underlying the apparent kidney-protective effect of tolvaptan. We propose that the preservation of the eGFRcr slope as observed in individuals who received tolvaptan compared with those who received placebo in landmark trials was an epiphenomenon of therapy-related loss of muscle mass. Selectively antagonizing the vasopressin receptor 2 with tolvaptan leads to polyuria ^6^, which can measure up to 10 liters per day ^8^. This requires excessive fluid consumption to match urine output. The ongoing need to urinate, coupled with the resulting thirst and fluids intake, adversely impacts quality of life. In addition, increased fluid consumption can reduce appetite ^3^, which, over time, may contribute to malnutrition and, consequently, gradual muscle loss. Loss of muscle mass reduces the total creatine pool, leading to lower creatinine production and circulating creatinine concentrations. Because circulating creatinine is inversely related to eGFRcr, therapy-related muscle loss can bias eGFRcr upward, irrespective of actual changes in GFR ^12^. This spurious elevation may explain the apparent preservation of eGFRcr in the tolvaptan group. Reevaluation of its kidney-protective effect using existing cystatin C data could yield important insights for patient care. eGFRcr, creatinine-based estimated glomerular filtration rate.

Autosomal dominant polycystic kidney disease (ADPKD) is an inherited systemic disorder affecting approximately 10% of patients receiving kidney replacement therapy ^1^. The disease is characterized by the formation of numerous fluid-filled cysts ( Figure 1), which already begin to develop in utero. Despite this early onset, most individuals remain asymptomatic for several decades. Over time, cyst growth causes increased kidney volume, irreversible disruption of the kidney parenchyma, and ultimately a relentless progression toward kidney failure, creating a constant source of uncertainty and distress for affected families ^1, 2^. In this context, the introduction of tolvaptan, a selective vasopressin receptor 2 antagonist, represented a significant milestone as the first—and currently the only—approved treatment shown to slow disease progression in ADPKD. Clinical trial data suggested that tolvaptan could reduce kidney growth by nearly 50% and slow the rate of kidney function decline by approximately 26% over a three-year period ^3^. This supported its approval for ADPKD in multiple countries, including Australia, Canada, Europe, and Japan. However, the US Food and Drug Administration (FDA) initially withheld approval due to concerns about potential liver injury and high dropout rates in the tolvaptan group, raising doubts about the reliability of the trial data and the true extent of benefit on kidney function ^4^. The Replicating Evidence of Preserved Renal Function: An Investigation of Tolvaptan Safety and Efficacy (REPRISE) trial addressed these concerns, showing that tolvaptan slowed the decline in estimated glomerular filtration rate (eGFR) by 1.27 ml/min per 1.73 m ^2^ compared with placebo ( Figure 1). The FDA thereafter approved tolvaptan, but concerns about its long-term efficacy remain ^5, 6^.

In clinical practice, the drug’s protective effect on kidney function—delaying progression to kidney failure in a patient with an eGFR of 41 ml/min per 1.73 m ^2^ with an average of 2.8 years (from 6.2 to 9.0 years) ^7^—must be balanced against its most burdensome side effect: polyuria of up to 10 liters per day ^6, 8^. Despite additional challenges such as disrupted sleep due to nightly urinating and a constant need of drinking water, initial reports indicated that 90% of patients accepted the treatment, often motivated by witnessing ADPKD complications in relatives ^6^. However, real-world data reveal a more nuanced picture. While tolerability within the first year of tolvaptan use aligns with clinical trial findings ^9, 10^, discontinuation rates rise substantially after three years ^10, 11^. This suggests that long-term adherence to tolvaptan may be more challenging than early trials implied.

An even more striking insight from real-world data is the observation that a substantial proportion of high-risk patients identified as candidates for tolvaptan never initiate treatment. Over 57% of eligible patients in the UK and 69% in Canada choose not to initiate therapy ^11, 13^. The anticipated impact of tolvaptan’s aquaretic effect on daily life, requiring up to 10 liters of fluid intake to match urine output ^8^, is cited as the primary reason for this reluctance ^13^. While patients who tolerate tolvaptan beyond three months often report minimal impact on quality of life, the experience of those unable to tolerate the drug remains unexplored. Similarly, no studies have examined the potential psychological or emotional burden on patients who decline treatment ^14^. For these individuals, and perhaps their families, the inability to adhere to a therapy that could delay kidney failure may lead to feelings of guilt or self-blame, negatively impacting their quality of life. This underscores the importance of rigorously testing hypotheses that challenge the kidney-protective effects observed in clinical trials. Beyond scientific truth-seeking, such efforts have profound humanistic implications, addressing not only the financial and physical costs of treatment, but also the psychological toll on patients unable to tolerate or unwilling to undergo therapy.

A critical aspect of evaluating tolvaptan’s efficacy lies in understanding potential adverse effects on muscle mass and the resulting confounding of kidney function surrogates ^12^. The Tolvaptan Efficacy and Safety in Management of Autosomal Dominant Polycystic Kidney Disease and Its Outcomes (TEMPO)3:4 and REPRISE trials assessed tolvaptan’s effect on kidney function by measuring changes in creatinine and creatinine-based eGFR (eGFRcr) ^3, 7^. Importantly, both the European Medicines Agency (EMA) and FDA recommend excluding the possibility of treatment-related changes in muscle mass before evaluating treatment effects on eGFRcr in clinical trials, because such changes could alter creatinine concentrations irrespective of actual changes in GFR and hence confound eGFRcr values ^15, 16^. Neither the TEMPO3:4 nor the REPRISE trial explicitly reported whether this recommendation was followed or whether treatment-related muscle mass changes were assessed and excluded ^3, 7^.

The substantially increased urinary output caused by tolvaptan induces thirst and leads to excessive fluid intake ( Figure 1). This increased fluid consumption can suppress appetite—a possibility supported by the higher incidence of anorexia reported with tolvaptan treatment compared with placebo ^3^. Appetite suppression preludes malnutrition and, over time, sarcopenia, reducing creatinine concentrations ^8, 17^ ( Figure 1). Additionally, fatigue resulting from disrupted sleep and frequent urination may discourage physical activity, further contributing to the progressive loss of muscle mass, which, in turn, lowers basal metabolic rate. Although body weight remained stable during the TEMPO3:4 trial ^3^, this does not exclude the possibility that the observed effects of tolvaptan on the change in kidney function were influenced by a reduction in muscle mass. As muscle mass and metabolic rate decline, the body enters a new steady state in which muscle mass stabilizes at a lower level. While the initial muscle loss following treatment initiation may be substantial, it does not continue indefinitely, but plateaus as the body adjusts over time.

Based on the trial data, a yearly muscle mass decline of approximately 1.3% would be sufficient to account for the observed effects on eGFRcr ^5, 12^. In a male with 35 kg of muscle, this equates to a loss of 0.46 kg per year, while in a female with 25 kg of muscle, this equates to 0.33 kg per year ^12^. If body weight remained stable, such muscle loss would imply an increase in fat mass of approximately 1.5 kg in males and 1.0 kg in females over three years. This scenario is plausible given the reduced physical activity and a lowered basal metabolic rate, coupled with the potential consumption of sugar-sweetened beverages to meet the substantially increased fluid requirements ^18, 19^.

When drug-related changes in muscle mass cannot be excluded, the EMA and FDA recommend using kidney function markers insensitive to variations in muscle mass, such as cystatin C, to evaluate eGFR over time in clinical trials ^15, 16^. Creatinine clearance was not suggested as an alternative ^15, 16^, likely due to the logistical challenges of 24-hour urine collection in clinical trials. Furthermore, patients on tolvaptan willing to complete such collections may represent a subset that would not be generalizable to the broader ADPKD population ^14^. While the use of directly measured GFR with exogenous filtration markers is mentioned as an alternative in the EMA and FDA recommendations ^15, 16^, such direct measurements are notoriously impractical and expensive. Therefore, analyses with cystatin C-based eGFR may provide meaningful insights. Although cystatin C concentrations are potentially more sensitive to factors such as inflammation, glucocorticoid exposure, and adiposity than creatinine ^20^, they remain a highly relevant marker for evaluating the effects of tolvaptan on kidney function ^21^, particularly in the early stages of ADPKD ^22^.

In the TEMPO3:4 trial, cystatin C was identified as a pharmacodynamic endpoint alongside urinary osmolality and urinary monocyte chemotactic protein-1 (MCP-1) concentrations ^23^. While findings on the effect of tolvaptan on osmolality and MCP-1 have been published ^24, 25^, findings on its effect on cystatin C as alternative marker have not been reported. The reason for this omission is unclear, but further evaluation is imperative. Not only would such reporting strengthen confidence in the drug’s kidney-protective effects—currently undermined by its likely adverse effect on muscle mass—it would also match evidence synthesis with regulatory recommendations. Above all, it is essential to exclude the potential confounding effect of a small muscle mass-lowering effect of tolvaptan on trial conclusions from the perspective of the emotional and psychological burdens borne by some patients and their families.

We hypothesize that a reevaluation of the TEMPO3:4 trial data using the available cystatin C measurements would reveal no significant effect of tolvaptan on slowing kidney function decline compared with placebo ( Figure 1). Moreover, the small degree of muscle mass decline required to account for the observed benefit on change in kidney function compared with placebo raises the possibility that a detrimental effect of tolvaptan on kidney function may have been concealed. Such a concealed detrimental effect stems from the drug’s aquaretic effects, which causes volume depletion or hypertonicity if treatment is not intermittently discontinued during episodes of inadequate fluid intake, vomiting, excessive sweating, or diarrhea ^26, 27^. Some patients may consequently experience faster disease progression due to emerging hypertonicity-related nephropathy ^27– 29^.

Testing this hypothesis would provide indispensable insights for patient care and healthcare systems. If confirmed, it may prompt reconsideration of tolvaptan’s role in managing ADPKD. More importantly, it would provide emotional relief for a potentially large population unable to tolerate or unwilling to start therapy due to its side effects, alleviating feelings of guilt and self-blame and freeing patients and their families from the psychological burden of declining a treatment believed to slow disease progression. Ultimately, these findings would reinforce the importance of robust validation for future therapies in nephrology, particularly where kidney-protective effects are anticipated.

## Ethics and consent

Ethics and consent is not required.

## Data Availability

No data are associated with this article.
