# Questioning the kidney-protective effects of tolvaptan: a testable hypothesis to revisit the current narrative

**DOI:** 10.12688/openreseurope.20428.2

**Published:** 2026-07-16

**Authors:** Dion Groothof, Nicole S. Erler, Stephan J.L. Bakker

**Affiliations:** 1 Internal Medicine, University Medical Center Groningen, Groningen, Groningen, 9713 GZ, The Netherlands; 2 Julius Center for Health Sciences and Primary Care, UMC Utrecht, Utrecht, The Netherlands

**Keywords:** ADPKD, Tolvaptan, Creatinine, Cystatin C, Muscle mass, Kidney function, Confounding

## Abstract

Tolvaptan is the only approved therapy for slowing disease progression in autosomal dominant polycystic kidney disease (ADPKD). Its use necessitates balancing kidney-protective effects against burdensome aquaretic side effects that limit long-term adherence. Both clinical and real-world data highlight that many eligible patients decline or discontinue therapy, complicating estimation of the true magnitude of kidney protection inferred from pivotal trials. In this perspective article, we hypothesise that two distinct sources of bias may influence estimates of treatment efficacy in opposite directions and explore the implications for the interpretation of trial results. First, we consider how treatment-related effects, particularly changes in creatinine generation, could bias creatinine-based estimated glomerular filtration rate (eGFR), potentially exaggerating the apparent benefit. Second, we evaluate how selective treatment discontinuation in the TEMPO3:4 trial may have distorted estimated treatment effects by altering which participants remain under observation over time, potentially attenuating the apparent benefit. Taken together, these considerations motivate a reassessment of placebo-adjusted treatment effects on eGFR using cystatin C measurements from TEMPO3:4, thereby enabling a within-trial, trajectory-based evaluation of potential treatment-induced changes in creatinine generation, supported by analytical approaches that better account for selective dropout. More broadly, our analysis extends beyond ADPKD, highlighting the need to re-evaluate existing clinical trials in which treatment may change creatinine generation, including those with sodium-glucose cotransporter-2 inhibitors and glucagon-like peptide-1 receptor agonists. Future trials should incorporate complementary filtration markers to assess treatment-related changes in creatinine generation. Across all trials, sensitivity analyses using contemporary modelling strategies are warranted to strengthen confidence in eGFR slope as a regulator-endorsed clinical endpoint. Improving the methodological rigour with which treatment effects are estimated is essential to ensure that clinical decisions accurately reflect therapeutic benefit relative to treatment burden and patient experience.

## Disclaimer

The views expressed in this article are those of the author(s). Publication in Open Research Europe does not imply endorsement of the European Commission.

## Introduction

**Figure 1.**
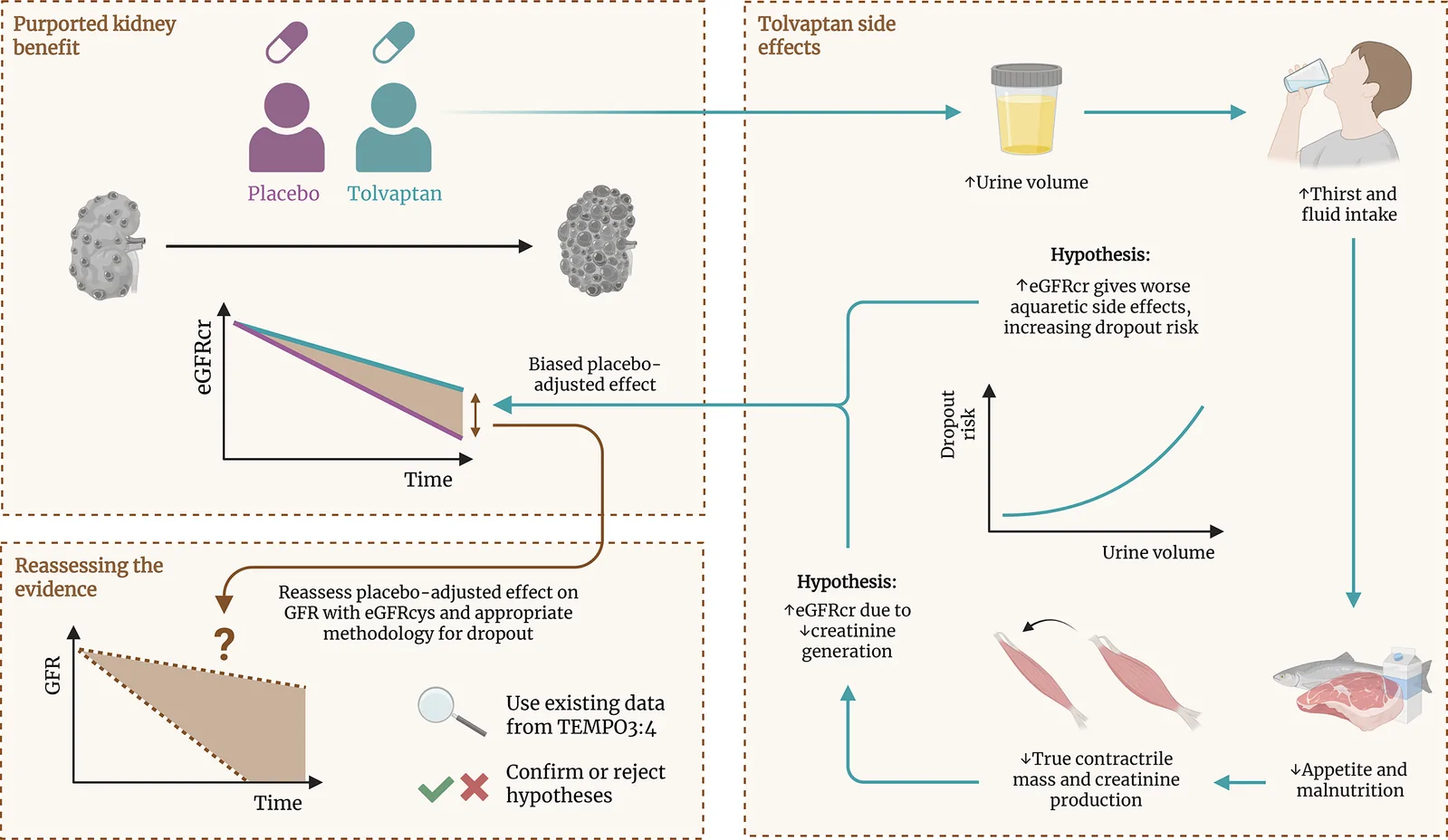
Hypothesized mechanism underlying the apparent kidney-protective effect of tolvaptan. We propose two distinct mechanisms through which tolvaptan therapy may have biased the estimated kidney-protective effects derived from eGFRcr in pivotal trials. First, selectively antagonising the vasopressin receptor 2 with tolvaptan leads to marked polyuria, ^6^ sometimes exceeding 12 litres per day, ^7^ necessitating substantial fluid consumption to match urine output. Increased fluid consumption may reduce appetite, ^3^ which, over time, can contribute to malnutrition and, consequently, gradual loss of metabolically active muscle mass. Loss of muscle mass reduces the total creatine pool, leading to lower creatinine generation and creatinine concentrations. Because creatinine concentrations are inversely related to eGFRcr, therapy-related reductions in creatinine generation may have led to an apparent preservation of eGFRcr upward, irrespective of actual changes in GFR, ^8^ thereby exaggerating the observed kidney-protective effect of tolvaptan. Second, higher urine volumes may also increase the risk of treatment discontinuation. Participants experiencing the most pronounced aquaretic effects, who may also have had more preserved baseline eGFRcr and higher urine output, ^9^ were therefore more likely to discontinue treatment and drop out prematurely. This selective attrition may have preferentially removed participants with more preserved eGFRcr from the tolvaptan arm, biasing the placebo-adjusted eGFRcr slope difference towards the null. Together, these biological and methodological pathways motivate a reassessment of the placebo-adjusted treatment effects on eGFRcr and eGFR derived from cystatin C, combined with analytical approaches that appropriately account for informative dropout. eGFRcr, creatinine-based estimated glomerular filtration rate.

Autosomal dominant polycystic kidney disease (ADPKD) is an inherited systemic disorder affecting approximately 10% of patients receiving kidney replacement therapy. ^1^ The disease is characterised by the formation of numerous fluid-filled cysts ( Figure 1), which already begin to develop in utero. Despite this early onset, most individuals remain asymptomatic for several decades. Over time, cyst growth causes increased kidney volume, irreversible disruption of the kidney parenchyma, and ultimately a relentless progression towards kidney failure, creating a constant source of uncertainty and distress for affected families. ^1, 2^ In this context, the introduction of tolvaptan, a selective vasopressin receptor 2 antagonist, represented a significant milestone as the first—and currently the only—approved treatment shown to slow disease progression in ADPKD. Clinical trial data suggested that tolvaptan could reduce kidney growth by nearly 50% and slow the rate of decline in estimated glomerular filtration rate (eGFR) by approximately 26% over a three-year period. ^3^ This supported its approval for ADPKD in multiple countries, including Australia, Canada, Europe, and Japan. However, the US Food and Drug Administration (FDA) initially withheld approval due to concerns about potential liver injury and high dropout rates in the tolvaptan group, raising doubts about the reliability of the trial data and the true extent of benefit on kidney function preservation. ^4^ The Replicating Evidence of Preserved Renal Function: An Investigation of Tolvaptan Safety and Efficacy (REPRISE) trial addressed these concerns, showing that tolvaptan slowed the decline in eGFR by 1.27 ml/min per 1.73 m ^2^ compared with placebo ( Figure 1). The FDA thereafter approved tolvaptan, but concerns about its long-term efficacy remain. ^5, 6^ We share these concerns and hypothesise that the observed kidney benefits of tolvaptan were, at least in part, confounded by therapy-related muscle loss, leading to an artefactual appearance of preserved GFR with tolvaptan. At the same time, there is reason to suspect that participant dropout in the pivotal trials was informative, raising the possibility that the observed treatment effects on change in eGFR were in fact underestimations of effects on true GFR. Together, these opposing yet plausible sources of bias highlight substantial uncertainty regarding both the direction and true magnitude of tolvaptan’s effect on GFR preservation. A reassessment of its impact on change in eGFR using contemporary analytical approaches capable of disentangling biological signal from measurement artefact and trial-related bias is therefore warranted.

This article substantiates the necessity of such a reassessment and is structured to provide a comprehensive and integrated evaluation of the biological and methodological factors that may have influenced the observed effects of tolvaptan on kidney disease progression in ADPKD. We first revisit the benefit-burden balance of tolvaptan therapy to establish the clinical context in which treatment decisions are made. We then examine mechanisms through which treatment-related effects could have influenced eGFR slope estimates in pivotal trials, and outline analytical approaches to evaluate and either confirm or exclude the presence of such effects. Next, we address a second potential source of bias by examining how missing follow-up data and treatment discontinuation arise in longitudinal studies and why standard analytical approaches may be compromised when discontinuation is related to unobserved outcomes, with specific reference to the design and analysis of past tolvaptan trials. Finally, we conclude with a structured synthesis that considers the implications for the interpretation of past, ongoing, and future trials and provide actionable directions for future research.

## Benefit-burden balance of tolvaptan

In clinical practice, the drug’s protective effect on kidney function—delaying progression to kidney failure, on average, from 6.2 to 9.0 years ^10^—must be balanced against its most burdensome side effect: polyuria of up to 12 litres per day. ^6, 7, 11^ Despite additional challenges such as disrupted sleep due to nightly urinating and a constant need of drinking water, initial reports indicated that 90% of patients accepted the treatment, often motivated by witnessing ADPKD complications in relatives. ^6^ However, real-world data reveal a more nuanced picture. While tolerability within the first year of tolvaptan use aligns with clinical trial findings, ^12, 13^ discontinuation rates rise substantially after three years. ^11, 13^ This suggests that long-term adherence to tolvaptan may be more challenging than early trials implied.

An even more striking insight from real-world data is the observation that a substantial proportion of patients at high risk of rapid disease progression identified as candidates for tolvaptan never initiate treatment. Over 57% of eligible patients in the UK and 69% in Canada choose not to initiate therapy. ^11, 14^ The anticipated impact of tolvaptan’s aquaretic effect on daily life, requiring up to 12 litres of fluid intake to match urine output, ^7, 11^ is cited as the primary reason for this reluctance. ^14^ While patients who tolerate tolvaptan beyond three months often report minimal impact on quality of life, the experience of those unable to tolerate the drug remains unexplored. Similarly, no studies have examined the potential psychological or emotional burden on patients who decline treatment. ^15^ For these individuals, and perhaps their families as well, the inability to adhere to a therapy that could delay kidney failure may lead to feelings of guilt or self-blame, negatively impacting their quality of life. This underscores the importance of rigorously testing hypotheses that challenge the kidney-protective effects observed in clinical trials.

## Muscle loss-related confounding of creatinine-based eGFR

A critical aspect of evaluating tolvaptan’s efficacy lies in understanding potential adverse effects on muscle mass and the resulting confounding of kidney function surrogates. ^7^ The Tolvaptan Efficacy and Safety in Management of Autosomal Dominant Polycystic Kidney Disease and Its Outcomes (TEMPO)3:4 and REPRISE trials assessed tolvaptan’s effect on kidney function by measuring changes in creatinine and creatinine-based eGFR. ^3, 10^ Creatinine is formed by the spontaneous, non-enzymatic dehydration of creatine and phosphocreatine, which are predominantly present in skeletal muscle. ^16^ Consequently, creatinine concentrations are directly determined by whole-body creatine pools and thus closely related to muscle mass. Once released into the circulation, creatinine is cleared almost exclusively by glomerular filtration, such that its steady-state concentration is inversely proportional to the GFR. This property makes creatinine a useful surrogate marker of GFR, provided that variation in creatinine production due to differences in muscle mass is adequately accounted for. Because muscle mass systematically varies with demographic characteristics, declining with advancing age and being, on average, lower in females than in males, creatinine concentrations also vary with age and sex independent of GFR. Estimating equations for GFR incorporate demographic variables in an attempt to account for this variability in creatinine generation, thereby providing a creatinine-based eGFR (eGFRcr) that more accurately reflects true GFR than creatinine concentrations alone. ^17^ Importantly, in light of the relationship between creatinine concentrations and muscle mass, both the European Medicines Agency (EMA) and FDA recommend excluding the possibility of treatment-related changes in muscle mass before evaluating treatment effects on eGFRcr in clinical trials, because such changes could alter creatinine concentrations irrespective of actual changes in GFR and hence confound eGFRcr values. ^18, 19^ Neither the TEMPO3:4 nor the REPRISE trial explicitly reported whether this recommendation was followed or whether treatment-related muscle-mass changes were assessed and excluded. ^3, 10^

Against this background, it is important to consider whether tolvaptan itself could plausibly influence muscle mass or creatinine generation through its known side-effect profile. The substantially increased urinary output caused by tolvaptan induces thirst and leads to excessive fluid intake ( Figure 1). To maintain fluid balance under these conditions, patients may preferentially consume palatable beverages rather than plain water, particularly when required volumes exceed several litres per day. Such choices can increase intake of sugar-sweetened or calorie-containing beverages. Regardless of how increased fluid intake is achieved, the markedly increased fluid consumption itself can suppress appetite—a possibility supported by the higher incidence of anorexia reported with tolvaptan treatment compared with placebo. ^3^ Appetite suppression may precede malnutrition and, over time, lead to muscle-mass loss and reduced creatinine generation, reducing blood creatinine concentrations independent of GFR ( Figure 1). ^7, 20^ Additionally, fatigue resulting from disrupted sleep and frequent urination may discourage physical activity, further contributing to the progressive muscle loss and a concomitant reduction in metabolic rate. Although body weight remained stable during the TEMPO3:4 trial, ^3^ this does not exclude the possibility that the observed effects of tolvaptan on changes in eGFRcr were influenced by reductions in muscle mass or, alternatively, by reductions in creatinine generation occurring despite preserved muscle mass. This distinction is crucial, because it is changes in creatinine generation, not muscle mass per se, that can bias eGFRcr. In both instances where metabolically active muscle mass and metabolic rate decline, the body enters a new steady state in which muscle mass stabilises at a lower level. Any initial muscle loss following treatment initiation would accordingly be expected to plateau over time rather than progress indefinitely. Importantly, even a transient reduction in creatinine generation early after treatment initiation would establish a new state of lower creatinine concentrations. While similar effects could in principle occur later during treatment, early changes are particularly relevant because they would persist throughout follow-up and thereby shift entire longitudinal eGFR trajectories rather than producing only transient deviations at individual time points.

To assess whether such changes could have meaningfully influenced trial outcomes, it is crucial to consider the magnitude of muscle loss that would be required to account for the treatment effect observed in the TEMPO3:4 trial. ^3^ A quantitative estimation of the degree of muscle-mass loss that could plausibly underlie the observed treatment effect on eGFRcr is provided in **Box S1** of the **Supplementary Appendix**. ^21^ Assuming that the reported between-group difference in the annual eGFRcr slope (estimated at −0.98 ml/min/1.73 m ^2^) was entirely attributable to reduced endogenous creatinine generation rather than true preservation of GFR, back-calculation from the trial data ^3^ indicates that a yearly decline in creatinine generation of approximately 1% would be sufficient. In absolute terms, this corresponds to a loss of approximately 0.33 kg of skeletal muscle per year in a typical male with 31.6 kg of muscle, ^22^ assuming a body weight equal to the mean body weight in the tolvaptan arm. If body weight were to remain stable, this degree of muscle loss would necessarily be accompanied by a compensatory increase in fat mass of approximately 1 kg over the 3-year follow-up period of TEMPO3:4. ^3^ Such a pattern is biologically plausible in the context of reduced physical activity, a lowered metabolic rate, and increased consumption of sugar-sweetened beverages to meet the substantially increased fluid requirements associated with tolvaptan therapy. ^23, 24^

These considerations underscore that even modest, clinically plausible changes in muscle mass or muscle composition could meaningfully influence eGFRcr. Importantly, if such changes were sufficient to account for the apparent GFR preservation observed in the TEMPO3:4 trial, ^3^ they also raise the possibility that a detrimental effect of tolvaptan on true GFR may have been concealed. In particular, the drug’s aquaretic effects may predispose susceptible patients to episodes of volume depletion or hypertonicity during intercurrent illness (e.g., vomiting, excessive sweating, or diarrhoea) or during periods of inadequate fluid intake if treatment is not intermittently discontinued. ^25, 26^ Some patients may consequently experience faster disease progression due to emerging hypertonicity-related nephropathy. ^26– 28^

While episodes of volume depletion or hypertonicity could exacerbate kidney injury, the frequency, severity, and clinical impact of such events in tolvaptan-treated patients with ADPKD remain undefined. At present, only indirect evidence from heat-stress nephropathy is available, ^26– 28^ which does not substitute for ADPKD-specific cohort data. Prospective monitoring of electrolytes, osmolality, and acute GFR changes during intercurrent illnesses would be necessary to substantiate this concern. Nonetheless, even this theoretical possibility of accelerated kidney injury underscores the need for analytical approaches capable of distinguishing true treatment effects on GFR from artefacts introduced by changes in creatinine generation.

## Approaches to evaluate potential confounding related to changes in muscle mass and creatinine generation in clinical trials

Given the plausible dissociation between body weight, muscle mass, and endogenous creatinine generation highlighted above, potential drug effects on muscle mass, muscle quality, and creatinine generation must be reliably assessed and either confirmed or confidently excluded. When a drug effect on true GFR is approximated by evaluating longitudinal changes in eGFRcr, creatinine generation must remain stable or any co-occurring changes must be appropriately accounted for. ^18, 19^

When evaluating the stability of creatinine generation over time during drug treatment, muscle mass and endogenous creatinine generation are often considered interchangeable, but this assumption does not always hold. Muscle mass and creatinine generation can be differentially affected by changes in muscle composition, including variations in intermyocellular fat (extracellular adipose tissue beneath the muscle fascia between myocytes), intramyocellular lipid content (lipid droplets within myocytes), and total muscle water content. In situations where a drug introduces clear and concordant reductions in both total body fat and muscle mass, changes in creatinine generation are likely to closely track changes in muscle mass. Notable examples are clinical trials with inhibitors of sodium-glucose cotransporter-2, as well as the glucagon-like peptide-1 (GLP-1) receptor analogue semaglutide, the glucose-dependent insulinotropic polypeptide (GIP) and GLP-1 receptor agonist tirzepatide, and the GIP, GLP-1, and glucagon receptor agonist retatrutide. ^29– 34^ Under these circumstances, standard techniques that primarily assess muscle mass rather than total body creatine pools, including magnetic resonance imaging (MRI), computed tomography scanning, dual-energy X-ray absorptiometry, bio-electrical impedance analysis, and muscle ultrasonography, can be used to estimate the magnitude of changes in muscle mass and, by extension, creatinine generation. ^35– 37^

By contrast, a different scenario arises when a drug reduces whole-body creatinine generation independent of measurable changes in muscle mass or volume. In such cases, muscle mass as assessed by standard techniques may appear unchanged, while creatinine generation has in fact decreased. Drugs with such properties that have been evaluated in the renal field include the synthetic glucocorticoids budesonide and methylprednisolone. ^38– 40^ Glucocorticoids are well known for their ability to induce muscle atrophy ^41^ alongside insulin resistance and a redistribution of body fat to promote fat infiltration of skeletal muscle. ^42^ Accordingly, glucocorticoid exposure may create a dissociation between apparent muscle mass and true contractile tissue content.

A similar situation could plausibly occur with tolvaptan. Reduced protein intake, decreased physical activity, and increased consumption of sugar-sweetened beverages to meet high fluid requirements could result in stable body weight while promoting a redistribution of fat from adipose tissue to intermuscular and intramyocellular compartments. This pattern converges on a muscle-composition phenotype that is well described in insulin-resistant states and in conditions associated with reduced physical activity. Under these conditions, muscle mass may appear preserved, while whole-body creatinine generation is nonetheless reduced. It is in this context that techniques that assess muscle mass without specifically quantifying total body creatine pools or creatinine generation are insufficient to exclude a clinically relevant drug effect on creatinine generation. Instead, methods capable of directly assessing total body creatine pools or creatinine generation are required. Among imaging techniques, only MRI Dixon (to quantify muscle volume) combined with ^1^H-magnetic resonance spectroscopy (for intramyocellular lipid and intramuscular creatine concentration) and ^31^P-magnetic resonance spectroscopy (for intramuscular phosphocreatine concentration) allow comprehensive assessment of the total endogenous creatine pool. ^43– 46^

## Detection of a clinically relevant drug effect on creatinine generation in clinical trials

Although the magnitude of change in creatinine generation required to confound eGFRcr is small and biologically plausible, confidence in the interpretation of creatinine-based trial outcomes depends on whether such changes can be empirically detected or confidently excluded using feasible study designs of relatively short duration. The yearly decline in creatinine generation of approximately 1% that would be sufficient to explain the observed treatment effect on eGFRcr in TEMPO3:4 corresponds to detectability of a 0.5% change in creatinine generation during a six-month study.

Translating this detectability threshold into a sample size estimate for a study requires assumptions regarding both the baseline size of the total endogenous creatine pool and the variability with which treatment-induced changes in that pool can be measured over time. Below, we focus on techniques that allow direct or near-direct quantification of changes in endogenous creatinine. To illustrate feasibility, we present a single condensed sample size calculation, while summarising estimates for alternative approaches. We show that required sample sizes vary substantially depending on the method used to assess creatinine generation and refer to the **Supplementary Methods** ^21^ for full derivations, methodological assumptions, and sample size calculations.

Reproducibility studies combining Dixon MRI with ^1^H- and ^31^P-magnetic resonance spectroscopy suggest that total-body creatine pool size, used as a proxy for creatinine generation, can be measured with relatively low within-subject variability across repeated measurements. ^43– 46^ Assuming a baseline pool of 135 g in a 79 kg male ^16^ and a standard deviation of 1.0 g for individual baseline-to-6-month changes, a 0.5% difference between treatment groups over six months corresponds to an absolute difference of 0.675 g. Under these assumptions, a total sample size of 70 participants (35 per group) would be required to detect such a difference with 80% power at a two-sided α of 0.05 in an independent-samples comparison of the mean change in creatine pool between placebo and intervention arms.

Direct isotope dilution techniques, while providing near-direct quantification of total body creatine pool size, are subject to greater variability and would require larger samples (approximately 250 participants in total). An indirect, regulator-endorsed approach to assessing treatment-related changes in creatinine generation is the use of alternative filtration markers that are less dependent on muscle mass, such as cystatin C. ^18, 19^ Because cystatin C is produced at a relatively constant rate by all nucleated cells, ^47^ independent of creatinine generation, regulatory agencies recommend its use as a confirmatory marker when drug-related effects on creatinine generation cannot be excluded. Comparison of creatinine-based eGFR and cystatin C-based eGFR (eGFRcys) therefore provides a practical means to detect divergence attributable to non-GFR determinants, including creatinine generation. However, approaches based on longitudinal discordance between eGFRcr and eGFRcys, although operationally simple and readily implementable in clinical trials, would require considerably larger sample sizes (on the order of several thousand participants) to detect similarly small changes over short time intervals. Lastly, estimates based on 24-hour urinary creatinine excretion fall between imaging-based and isotope dilution-based approaches (approximately 150 participants under ideal conditions), but are highly sensitive to collection error in unsupervised, free-living populations. This would likely necessitate substantially larger sample sizes in contemporary clinical trials.

Although evaluation of discordance between eGFRcr and eGFRcys trajectories requires substantially larger sample sizes than approaches based on 24-hour urinary creatinine excretion under ideal conditions, it offers a unique practical advantage. Because creatinine is already measured repeatedly to derive eGFRcr trajectories in clinical trials, cystatin C can be measured in the same blood samples, allowing longitudinal discordance between eGFRcr and eGFRcys to be evaluated at relatively low cost, without additional participant burden or the need for a separate confirmatory study. In this framework, treatment-induced changes in creatinine generation can be evaluated by testing whether the placebo-adjusted trajectory of eGFRcr diverges over time from the corresponding trajectory of eGFRcys.

In patients with ADPKD, eGFRcr and eGFRcys are moderately correlated, sharing approximately 30% of their variance, ^48^ consistent with their common dependence on true GFR. The remaining variance attributable to non-GFR determinants of each marker, including creatinine generation for eGFRcr, provides the statistical basis for detecting treatment-induced divergence between the two markers over time. In TEMPO3:4, creatinine as a secondary efficacy endpoint and cystatin C as a prespecified pharmacodynamic variable were both defined in the trial protocol (Supplementary Material of the original report), ^3^ allowing derivation of repeated measurements of eGFRcr and eGFRcys and, in principle, assessment of the divergence between these estimates. Given the longitudinal structure and sample size of the trial, such a within-trial, trajectory-based approach would have allowed detection of a minimum cumulative treatment-induced change in endogenous creatinine generation of approximately 1.2% over three years ( **Supplementary Methods S6** ^21^). This estimate assumes a conservative correlation of 0.3 between longitudinal changes in eGFRcr and eGFRcys, allowing for progressive discordance due to time-varying non-GFR determinants of each marker, and is anchored to the number of participants remaining under observation at three years in TEMPO3:4. ^3^ Because detection of such a cumulative change corresponds to resolving an average change of approximately 0.2% per six months, this approach offers substantially greater statistical power and practical feasibility than conducting a separate short-term study with two repeated measurements.

Taken together, these considerations indicate that therapy-induced changes in creatinine generation of clinically relevant magnitude are not only biologically plausible but also empirically detectable within existing trial datasets. However, whether such signals can be validly interpreted depends critically on the assumptions underlying models for longitudinal data when follow-up is incomplete.

## Missing data, informative dropout, and model validity

The primary analytical approach used in the TEMPO3:4 trial relied on linear mixed-effects models. ^3^ While such models are widely used in clinical trials with repeatedly measured continuous outcomes, their validity depends on several assumptions, including that missing outcome data are missing at random (MAR). ^49– 51^ Here, ‘missing data’ refers to the absence of measurements originally scheduled as part of a study, a situation that commonly occurs in clinical research due to factors such as protocol-defined measurement discontinuation, treatment cessation, withdrawal of consent, intercurrent illness, loss to follow-up including death, and logistical or administrative constraints. Under the MAR assumption, the probability that a measurement is missing depends only on information that has already been observed and is included in the model (i.e., covariates or previously observed longitudinal values). Examples of MAR in nephrology include situations in which follow-up eGFR measurements are discontinued after a patient’s eGFR exceeds a predefined safety threshold, or when biomarker testing is purposefully omitted because levels of another marker fall within expected ranges. A notable example is the omission of cystatin C testing when a patient’s muscle mass, serving as a proxy of creatinine generation, is considered typical. In such situations, the missing-data mechanism can be considered MAR only if the variable driving the decision to omit testing (e.g., muscle mass or its proxy) is explicitly included in the model. More generally, missingness may arise from protocol-defined criteria or from clinical decision-making based on prior information. In practice, this means that relevant observed measurements that inform the missingness (e.g., prior eGFR or related biomarkers) should be included in the modelling approach. When this assumption holds, linear mixed-effects models can provide valid inference for longitudinal treatment effects. ^49^

A distinct situation arises when the probability that a measurement is missing depends on information that has not been observed, in which case missing outcome data are missing not at random (MNAR). ^49^ In clinical trials, this may occur when patients discontinue treatment because of worsening symptoms or declining eGFR that is not fully captured in prior measurements. In such cases, dropout is informative, with missing data depending directly on unobserved outcomes that would have been measured had follow-up continued. As a result, patients who leave the study prematurely differ systematically from those who remain, increasing the risk that analyses assuming MAR yield biased estimates of treatment effects. ^49^

In practice, dropout mechanisms may reflect a combination of MAR and MNAR processes, with different factors operating simultaneously or at different stages of follow-up. Although such mixed mechanisms have been examined in methodological work, ^52– 54^ their detailed consideration is beyond the scope of the present discussion. Regardless of the exact situation, the magnitude and even the direction of the ensuing bias in the estimation of eGFR slope depend critically on how and why follow-up data become missing. For this reason, contemporary methodological guidance, aligned with current FDA recommendations, emphasises that sensitivity analyses are an indispensable component of longitudinal trial analysis when inference relies on missing-data assumptions. ^55, 56^ This emphasis is grounded in the recognition that, as clearly noted in the work of Rizopoulos, the observed data alone cannot distinguish between MAR and MNAR dropout mechanisms, making sensitivity analyses the only pragmatic way to evaluate the impact of potential violations of these assumptions. ^51^

The extent to which such analyses can be effectively performed varies across the pivotal ADPKD trials. Although the REPRISE trial permitted partial evaluation of missing-data assumptions within an intention-to-treat framework, as follow-up measurements of eGFRcr continued after treatment discontinuation, it does likely not allow assessment of potential divergence of trajectories of eGFRcr and eGFRcys. This is because, unlike the TEMPO3:4 trial, measurement of cystatin C was not specified in the trial protocol (Supplementary Material of the original report). ^10^ As a result, sensitivity analyses that jointly address missing-data assumptions and distinguish effects on true GFR from artifacts related to treatment-induced changes in endogenous creatinine generation would require post-hoc cystatin C assessment in stored samples.

In contrast to REPRISE, the design of the TEMPO3:4 trial imposes more fundamental constraints on analogous sensitivity analyses. In TEMPO3:4, follow-up measurements of eGFRcr were, by design, discontinued when treatment was stopped, ^3, 57^ precluding observation of eGFRcr trajectories beyond the point of discontinuation. This discrepancy with the intention-to-treat framework employed in REPRISE is important, because it places greater reliance on assumptions about unobserved eGFRcr values after discontinuation. In models fit under a MAR assumption, including standard linear mixed-effects models, missing post-discontinuation eGFRcr values are implicitly extrapolated from each participant’s model-estimated trajectory based on their observed data. In effect, this amounts to assuming that, conditional on the data observed up to the point of discontinuation, participants who stop treatment would, on average, have continued to follow the same eGFRcr trajectory as those who remain under observation. Admittedly, the plausibility of this assumption becomes questionable when the reasons for treatment discontinuation are related to a participant’s underlying kidney function trajectory and are not fully captured by the observed data included in the model. In such situations, the assumption that participants who discontinue would, on average, have continued to follow the same trajectory as those remaining under observation may no longer be tenable. Frequent discontinuation and systematic between-group differences may further amplify the impact of such mechanisms, although these features alone do not establish informative (MNAR-type) dropout. This motivates closer examination of the extent and determinants of treatment discontinuation in TEMPO3:4.

In that trial, treatment discontinuation occurred in 221 of 961 participants (23.0%) assigned to tolvaptan compared with 67 of 483 participants (13.8%) assigned to placebo (χ ^2^ = 16.8; P < 0.0001). ^3^ This pronounced imbalance between the two groups suggests that leaving the study may have been linked to effects or side-effects of the treatment, even if the underlying reasons for discontinuation were similar across groups but occurred more frequently in the tolvaptan arm. Combined with the possibility that reasons for leaving the study may not be fully captured by the observed data, this raises the possibility of an MNAR-type mechanism in the context of missing-data analysis.

A clinically plausible explanation for the observed differential discontinuation relates to the side-effect profile of tolvaptan. The drug’s aquaretic effects, particularly severe polyuria, are a leading cause of rapid treatment discontinuation, as consistently documented in real-world cohorts. ^11, 14^ Similar patterns were observed in TEMPO3:4, where aquaresis-associated adverse effects were reported by more than half of tolvaptan-treated participants and were the commonest reason for treatment discontinuation. ^3^ Importantly, urine volume during tolvaptan treatment is not randomly distributed across patients. Kramers et al. demonstrated that higher baseline measured GFR (determined using continuous infusion of ^125^I-iothalamate and ^131^I-hippuran) and higher eGFRcr were independently associated with higher urine volume during tolvaptan treatment. ^9^ Given that greater urine volume directly translates into more pronounced aquaretic symptoms, these observations suggest that participants with more preserved kidney function were more likely to experience intolerable aquaretic symptoms during tolvaptan treatment, thereby increasing the likelihood of treatment discontinuation and early study dropout ( Figure 1). Importantly, consistent with observations of increasing discontinuation rates with longer-term tolvaptan use, ^11, 13^ the burden of aquaretic symptoms may evolve over time and is only partially reflected in observed eGFR values, which themselves are subject to biological variability and measurement fluctuations. ^58– 60^ As a result, the probability of treatment discontinuation may depend on factors not fully captured by prior observed measurements, providing a plausible basis for MNAR-type dropout mechanisms.

In the context of longitudinal analyses based on linear mixed-effects models fitted under a MAR assumption, selective discontinuation of this kind has important implications for the estimation of eGFRcr slopes. Preferential discontinuation of tolvaptan among participants with higher baseline eGFRcr would shift the composition of the remaining cohort in the tolvaptan arm towards participants with lower eGFRcr and more advanced disease, thereby leading to a steeper estimated population-level decline in eGFRcr than would have been observed in the absence of selective dropout. In contrast, discontinuation in the placebo arm is more plausibly driven by disease progression, such that participants experiencing more rapid declines in kidney function are more likely to drop out. This mechanism would similarly shift the composition of the remaining cohort in the placebo arm towards participants with higher eGFRcr and slower progression, yielding an artificially flatter estimated eGFRcr slope. Together, these opposing selection processes could, if not fully captured by the previously observed eGFR measurements and covariates included in the model, bias the placebo-adjusted treatment effect towards the null ( Figure 1), resulting in underestimation of the true effect of tolvaptan on GFR decline.

A similar selection process may also help explain why the reported effect of tolvaptan on annual rate of total kidney volume growth, the primary endpoint of the TEMPO3:4 trial, appears largest early after treatment initiation and attenuates thereafter. ^3^ This temporal pattern has previously been discussed, particularly with respect to whether early reductions in total kidney volume growth necessarily translate into sustained effects on kidney function decline, while it has also been emphasised that such early structural changes may still reflect a meaningful disease-modifying mechanism with longer-term implications. ^5, 61^ Importantly, while the latter interpretation supports the biological plausibility of a beneficial effect of tolvaptan, it does not preclude the possibility that concurrent mechanisms unrelated to GFR, as well as selective treatment discontinuation, may bias estimates of treatment effect on eGFRcr. From this perspective, if informative dropout is present, conventional analyses may attenuate the true effect of tolvaptan on GFR decline.

At the same time, given that creatinine concentrations (and eGFRcr values derived from those) were measured at regular 4-month intervals in TEMPO3:4, ^3^ it remains uncertain to what extent these evolving symptom-driven mechanisms are fully captured by prior observed measurements, and hence whether the resulting dropout process materially violates a MAR assumption. As emphasized by Rizopoulos, ^51^ the observed data cannot distinguish between MAR and MNAR mechanisms, underscoring the need for sensitivity analyses to evaluate the impact of potential departures from this assumption on estimates of treatment effect. Such analyses are increasingly applied in contemporary renal trials, ^30, 34, 39^ including the FLOW trial evaluating the effect of semaglutide on kidney outcomes. ^30^ Notably, in that study, sensitivity analyses were reported for eGFRcr but not for eGFRcys, despite the latter being less frequently measured. This discrepancy is relevant, as less frequent assessment of cystatin C increases the likelihood that prior observed values do not fully capture the processes underlying dropout. As a result, the MAR assumption may be more readily violated for eGFRcys-based analyses, increasing the likelihood of MNAR-type mechanisms and, consequently, biased estimation of eGFR slope.

Importantly, the limitations of the pivotal trial design, arising from selective treatment discontinuation and reliance on unverifiable MAR assumptions inherent to linear mixed-effects modelling in the presence of missing outcome data, were not addressed in the TEMPO4:4 open-label extension trial. ^62^ Because enrolment into TEMPO4:4 was restricted to participants who completed TEMPO3:4, continued follow-up was conditioned on treatment persistence during the randomised phase. As a result, inclusion of the TEMPO4:4 extension data in the analyses ^62^ cannot be expected to mitigate informative dropout arising during the pivotal trial ^3^ and may instead reinforce attenuation of the estimated treatment effects.

Taken together, these considerations indicate that selective treatment discontinuation related to unobserved (parts of ) outcome trajectories may introduce bias into estimates derived from standard mixed-effects models fitted under a MAR assumption. In the setting of TEMPO3:4, the opposing selection mechanisms described above could, if not fully captured by the observed data, bias the placebo-adjusted treatment effect towards the null, leading to potential underestimation of the true treatment effect of tolvaptan on GFR decline. At the same time, because the observed data alone cannot distinguish between MAR and MNAR dropout mechanisms, it remains uncertain whether such bias materially affected the original analyses. This uncertainty underscores the importance of sensitivity analyses that both exploit creatinine and cystatin C to explicitly evaluate the impact of alternative assumptions regarding missing-data mechanisms.

## Concluding remarks and future directions

In this article, we examined the benefit-burden balance of tolvaptan therapy and reviewed mechanisms through which treatment-induced effects, most notably changes in endogenous creatinine generation, could have influenced eGFRcr slope estimates in pivotal trials. We highlighted that such changes may not only exaggerate apparent kidney-protective effects when GFR is assessed with eGFRcr, but also obscure a detrimental effect on true GFR. We discussed analytical approaches to evaluate and either confirm or exclude the presence of such effects, emphasising that a within-trial, trajectory-based approach leveraging existing data from TEMPO3:4 offers the greatest statistical efficiency and practical feasibility for this purpose. We further illustrated how missing follow-up data due to treatment discontinuation can compromise standard analyses when missingness is related to unobserved (parts of ) outcome trajectories. This concern was motivated by the observed imbalance in treatment discontinuation in TEMPO3:4 ^3^ and supported by plausible biological mechanisms.

Taken together, these considerations indicate that estimates of the placebo-adjusted eGFRcr slope difference may have been influenced by two distinct sources of bias, each acting through a different mechanism. First, treatment-related changes in endogenous creatinine generation may have reduced the apparent decline in eGFRcr in the tolvaptan arm, thereby exaggerating the observed kidney-protective effect. Second, selective, arm-specific treatment discontinuation may have preferentially removed participants with better kidney function and slower progression from the tolvaptan arm, while preferentially retaining slower progressors in the placebo arm, thereby potentially attenuating the observed kidney-protective effect.

Addressing these intertwined biological and methodological challenges requires analytical approaches that can disentangle potential treatment-induced changes in creatinine generation from true kidney-protective effects, while simultaneously accounting for treatment discontinuation and loss to follow-up. Re-analyses of trials on tolvaptan and other drugs that may influence creatinine generation, along with future clinical studies, should all consider the following analytical strategies:

• Joint models for longitudinal and time-to-event data, in which biomarker trajectories and time-to-treatment discontinuation are modelled simultaneously, allowing the dependence between biomarker evolution and discontinuation to be explicitly represented rather than assumed away. ^51^ When relevant, competing-risk structures, preferably using cause-specific hazards, ^63^ should be specified to distinguish different dropout or intercurrent event processes;
• Flexible longitudinal sub-models, including spline-based representations of time, to accommodate potential non-linearity in biomarker change over time and reduce the risk of mis-specifying population-level slopes ^49^;
• Explicit modelling of informative dropout and intercurrent events, including competing and mixed dropout mechanisms, thereby considering treatment discontinuation and related intercurrent events as outcomes of interest rather than as nuisance processes ^52– 54^;
• Parallel modelling of eGFRcr and eGFRcys, enabling direct evaluation of whether treatment effects differ by filtration marker and facilitating evaluation of potential treatment-induced changes in endogenous creatinine generation;
• Sensitivity analyses under alternative missing-data assumptions, to examine how estimated treatment effects change under plausible departures from MAR-type mechanisms and to characterise the range of effects compatible with the observed data.

These strategies can be implemented within either frequentist or Bayesian inferential frameworks. Fully Bayesian approaches are particularly attractive, as they allow longitudinal outcomes, dropout processes, and incomplete covariate information to be modelled jointly under a single coherent probability model. In this setting, imputation of missing covariate data is performed simultaneously with model estimation, ensuring compatibility between analysis and imputation models and appropriately propagating uncertainty due to missing data into all inferential quantities. Such approaches are now readily implementable using contemporary statistical software. ^64^

The foregoing considerations have immediate implications for the design and interpretation of ongoing and future clinical trials. The ongoing HYDRO-PROTECT trial ( ClinicalTrials.gov number, NCT05373264), which evaluates whether hydrochlorothiazide can be added to tolvaptan to mitigate aquaretic side effects without compromising efficacy, is directly addressing a key determinant of treatment discontinuation. ^65^ However, the primary analyses described in the trial protocol rely on linear mixed-effects models and cystatin C measurements have not been specified, thereby limiting the ability to evaluate treatment effects on creatinine generation or to apply joint modelling strategies of the type discussed here. Considering treatment discontinuation, the possibility of guilt and self-blame among patients who refuse to start or discontinue using tolvaptan currently remains largely conjectural, as qualitative or quantitative evidence on patient-reported emotional outcomes in this context is lacking. We recommend performance of studies (e.g., structured interviews, validated quality-of-life instruments) capable of capturing these dimensions.

More broadly, the issues highlighted here extend beyond tolvaptan and ADPKD. Trials across nephrology increasingly rely on eGFR slope as a regulator-endorsed surrogate endpoint, supported by extensive methodological evaluation and by its practical advantages for trial feasibility. ^18, 19, 66, 67^ At the same time, may contemporary interventions introduce precisely the complexities discussed in this article, including acute treatment effects on eGFR, substantial treatment discontinuation, informative missingness, and treatment-related changes in body composition that may alter creatinine generation independently of true GFR. These concerns are particularly relevant for trials of therapies associated with weight loss or altered muscle composition, including SGLT2 inhibitors and incretin-based therapies such as semaglutide, tirzepatide, retatrutide, and the oral non-peptide GLP-1 receptor agonist orfoglipron. ^29– 34, 68^ In these settings, kidney outcomes and kidney-related parameters are increasingly being evaluated using creatinine-based eGFR slopes. Careful consideration of missing-data mechanisms, incorporation of complementary filtration markers such as cystatin C, and application of sensitivity analyses under alternative missing-data assumptions should therefore be integral to trial design and analysis.

Ultimately, improving the methodological rigour with which treatment effects are estimated is not merely a statistical exercise. It directly informs the balance between therapeutic benefit and treatment burden experienced by patients. Ensuring that estimates of efficacy are as unbiased and interpretable as possible is therefore essential to guide clinical decision-making and to ensure that treatment decisions are grounded in robust evidence.

## Ethics and consent

Ethics and consent is not required.

## Data Availability

Supplementary material supporting this perspective article has been deposited in the Zenodo repository. ^21^ Repository name: Zenodo. Title of project: Supplementary Appendix: Quantitative Estimation of the Muscle-Mass Decline Required to Bias Creatinine-Based eGFR and Sample Size Considerations for Detecting Treatment-Induced Changes in Endogenous Creatinine Generation in Trials Using Creatinine-Based eGFR Outcomes. DOI: https://doi.org/10.5281/zenodo.21158706 The repository contains **Box S1**, which presents the quantitative estimation of the muscle-mass decline required to meaningfully bias creatinine-based eGFR. It also contains the **Supplementary Methods**, which describe the methodological assumptions, derivations, and sample size calculations related to detecting treatment-induced changes in endogenous creatinine generation in creatinine-based eGFR analyses. ^21^ The material is available under the terms of the Creative Commons Attribution 4.0 International license (CC BY 4.0).
