# Supplementary material for: Questioning the kidney-protective effects of tolvaptan: a testable hypothesis to revisit the current narrative
Source: Open Res Eur. 2026 Jul 16;5:152. Originally published 2025 Jun 4. [Version 2] doi: 10.12688/openreseurope.20428.2 (PMC13507848; doi:10.12688/openreseurope.20428.2)
Supplement: Peer review history

# Reviewer report: Reviewer response for version 2

**DOI:** 10.21956/openreseurope.26036.r77464

**Published:** 2026-08-18

**Authors:** Michiel Betjes

**Affiliations:** 1 Erasmus Medical Center, Rotterdam, The Netherlands

The authors have made substantial and valuable changes to the revised manuscript.

Where applicable, are recommendations and next steps explained clearly for others to follow? (Please consider whether others in the research community would be able to implement guidelines or recommendations and/or constructively engage in the debate)

Yes

Does the article adequately reference differing views and opinions?

Partly

Are all factual statements correct, and are statements and arguments made adequately supported by citations?

Yes

Is the rationale for the Open Letter provided in sufficient detail? (Please consider whether existing challenges in the field are outlined clearly and whether the purpose of the letter is explained)

Yes

Is the Open Letter written in accessible language? (Please consider whether all subject-specific terms, concepts and abbreviations are explained)

Yes

Reviewer Expertise:

general nephrology and kidney transplantation

I confirm that I have read this submission and believe that I have an appropriate level of expertise to confirm that it is of an acceptable scientific standard.

# Reviewer report: Reviewer response for version 2

**DOI:** 10.21956/openreseurope.26036.r77466

**Published:** 2026-07-25

**Authors:** Amaryllis van Craenenbroeck

**Affiliations:** 1 KU Leuven, Louvain, Belgium

Thank you for this revised version of the manuscript. The authors addressed all raised concerns.

Where applicable, are recommendations and next steps explained clearly for others to follow? (Please consider whether others in the research community would be able to implement guidelines or recommendations and/or constructively engage in the debate)

Yes

Does the article adequately reference differing views and opinions?

Yes

Are all factual statements correct, and are statements and arguments made adequately supported by citations?

Yes

Is the rationale for the Open Letter provided in sufficient detail? (Please consider whether existing challenges in the field are outlined clearly and whether the purpose of the letter is explained)

Yes

Is the Open Letter written in accessible language? (Please consider whether all subject-specific terms, concepts and abbreviations are explained)

Yes

Reviewer Expertise:

nephrology, vascular dysfunction and its reversibility by exercise training

I confirm that I have read this submission and believe that I have an appropriate level of expertise to confirm that it is of an acceptable scientific standard.

# Reviewer report: Reviewer response for version 2

**DOI:** 10.21956/openreseurope.26036.r77465

**Published:** 2026-07-21

**Authors:** Ellen Hoogeveen

**Affiliations:** 1 Clinical Epidemiology, Leiden University Medical Center, Leiden, The Netherlands

The Open Letter has been substantially improved and is now well considered, thoroughly thought out, and clearly presented. I have no further comments.

Where applicable, are recommendations and next steps explained clearly for others to follow? (Please consider whether others in the research community would be able to implement guidelines or recommendations and/or constructively engage in the debate)

Yes

Does the article adequately reference differing views and opinions?

Yes

Are all factual statements correct, and are statements and arguments made adequately supported by citations?

Yes

Is the rationale for the Open Letter provided in sufficient detail? (Please consider whether existing challenges in the field are outlined clearly and whether the purpose of the letter is explained)

Yes

Is the Open Letter written in accessible language? (Please consider whether all subject-specific terms, concepts and abbreviations are explained)

Yes

Reviewer Expertise:

nephrology and clinical epidemiology

I confirm that I have read this submission and believe that I have an appropriate level of expertise to confirm that it is of an acceptable scientific standard.

# Reviewer report: Reviewer response for version 1

**DOI:** 10.21956/openreseurope.22106.r55603

**Published:** 2025-07-15

**Authors:** Ellen Hoogeveen

**Affiliations:** 1 Clinical Epidemiology, Leiden University Medical Center, Leiden, The Netherlands

**Summary**

 Groothof and Bakker present a thought-provoking hypothesis that the apparent kidney-protective effect of tolvaptan in ADPKD trials may, at least partially, be attributed to therapy related muscle wasting - leading to lower serum creatinine and a spurious rise in creatinine-based eGFR (eGFRcr) - rather than true preservation of glomerular filtration. They propose that tolvaptan’s aquaretic effects (polyuria up to 10 L/day) drive excessive fluid intake, appetite suppression, malnutrition, and gradual sarcopenia, which in turn raises eGFRcr. The authors note that cystatin C - a filtration marker insensitive to muscle mass - was measured in TEMPO3:4 but never reported, and call for reanalysis of those data. They further speculate that intermittent hypertonic dehydration events may actually worsen kidney injury, and highlight the psychological burden on patients who decline or discontinue tolvaptan due to its side effects.

**Major Comments**

**1. Speculative muscle-mass loss and eGFR confounding**

 The central premise - that tolvaptan induces sufficient muscle wasting to alter creatinine pools and confound eGFRcr - is plausible but unsupported by direct longitudinal body-composition or nutritional data from trial participants. The extrapolation of a 1.3% annual muscle-mass decline derives from general sarcopenia literature rather than ADPKD cohorts on tolvaptan, and the TEMPO3:4 trial did not report serial lean-mass measurements or nutritional assessments to substantiate clinically meaningful sarcopenia.

**2. Absence of published cystatin C results**

 Although cystatin C was prespecified as a pharmacodynamic endpoint in TEMPO3:4, it is not clarified whether these data exist in full, nor discussed why they were omitted.[1] The authors of the TEMPO3:4 trial could investigate and report whether cystatin C trajectories differed between tolvaptan and placebo arms, and if not, whether non-significance or logistical constraints explain the absence of publication.

**3. Existing cystatin C literature in ADPKD**

 Several studies (e.g., Sans et al., 2017) have evaluated cystatin C-based eGFR in early ADPKD stages and found concordance with eGFRcr, suggesting limited creatinine bias in this setting.[2] The manuscript would be strengthened by situating the hypothesis within this existing body of work.

**4. Psychological burden claims need empirical backing**

 The discussion of guilt and self-blame among patients who forgo or stop tolvaptan is an important humanistic angle but remains conjectural. There is currently no published qualitative or quantitative research exploring patient-reported emotional outcomes in this context. The authors should recommend or initiate studies (e.g., structured interviews, validated quality-of-life instruments) to capture these dimensions.

**5. Hypertonicity-related nephropathy hypothesis**

 While episodes of volume depletion or hypertonicity could theoretically exacerbate kidney injury, the frequency, severity, and clinical impact of such events in tolvaptan-treated ADPKD patients remain undefined. Citing indirect evidence from heat-stress nephropathy does not substitute for ADPKD-specific cohort data. Prospective monitoring of electrolytes, osmolality, and acute GFR changes during intercurrent illnesses would be necessary to substantiate this concern.

**Minor Comments**

- **Body weight vs. composition**: The manuscript notes stable body weight during TEMPO3:4, and suggests that fat-mass increases could mask lean-mass loss; recommending DXA or bioimpedance analyses in future trials would be helpful.
- **Statistical framework**: Propose power calculations and minimal detectable difference for a cystatin C - based reanalysis to guide feasibility.
- **Figure clarity**: In Figure 1, consider annotating with estimated effect sizes (e.g., expected decline in creatinine per kg muscle lost) to aid interpretability.

**Conclusion**

 This perspective raises a biologically plausible challenge to the current narrative on tolvaptan’s kidney-protective effect and underscores important recommendations to account for muscle-mass changes. However, it remains speculative without direct empirical support. To advance the field, the authors could (1) propose to seek and publish the TEMPO3:4 cystatin C data; (2) propose prospective body-composition and patient-reported outcome measures, including a qualitative study; and (3) rigorously evaluate the hypertonicity hypothesis in clinical practice. Such efforts will ensure that decisions about tolvaptan use are grounded in robust, multifaceted evidence.

**References**

 1.Torres VE, Meijer E, Bae KT, *et al:* Rationale and design of the TEMPO(Tolvaptan Efficacy and Safety in Management of autosomal dominant

 polycystic kidney disease and its outcomes) 3-4 Study. *Am J Kidney Dis. *2011;

 57(5): 692–9. PMID: 21333426

 2. Sans *et al:* Cystatin C estimated glomerular filtration rate to assess renal function in early stages of autosomal dominant polycystic kidney disease *PLoS One.* 2017;12(3):e0174583. PMID: 28346513

Where applicable, are recommendations and next steps explained clearly for others to follow? (Please consider whether others in the research community would be able to implement guidelines or recommendations and/or constructively engage in the debate)

Yes

Does the article adequately reference differing views and opinions?

Yes

Are all factual statements correct, and are statements and arguments made adequately supported by citations?

Yes

Is the rationale for the Open Letter provided in sufficient detail? (Please consider whether existing challenges in the field are outlined clearly and whether the purpose of the letter is explained)

Yes

Is the Open Letter written in accessible language? (Please consider whether all subject-specific terms, concepts and abbreviations are explained)

Yes

Reviewer Expertise:

nephrology and clinical epidemiology

I confirm that I have read this submission and believe that I have an appropriate level of expertise to confirm that it is of an acceptable scientific standard, however I have significant reservations, as outlined above.

## References

[1] : Rationale and Design of the TEMPO (Tolvaptan Efficacy and Safety in Management of Autosomal Dominant Polycystic Kidney Disease and Its Outcomes) 3-4 Study. *American Journal of Kidney Diseases* .2011;57(5) : 10.1053/j.ajkd.2010.11.029692-699 10.1053/j.ajkd.2010.11.029 21333426

[2] : Cystatin C estimated glomerular filtration rate to assess renal function in early stages of autosomal dominant polycystic kidney disease. *PLOS ONE* .2017;12(3) : 10.1371/journal.pone.0174583 10.1371/journal.pone.0174583 PMC536770228346513

# Response

**Published:** 2026-05-17

**Authors:** Dion Groothof

**Affiliations:** Internal Medicine, University Medical Center Groningen, Groningen, Groningen, The Netherlands

**Detailed, itemised response to the comments by Reviewer Dr. Ellen Hoogeveen** **Summary** Groothof and Bakker present a thought-provoking hypothesis that the apparent kidney-protective effect of tolvaptan in ADPKD trials may, at least partially, be attributed to therapy related muscle wasting - leading to lower serum creatinine and a spurious rise in creatinine-based eGFR (eGFRcr) - rather than true preservation of glomerular filtration. They propose that tolvaptan’s aquaretic effects (polyuria up to 10 L/day) drive excessive fluid intake, appetite suppression, malnutrition, and gradual sarcopenia, which in turn raises eGFRcr. The authors note that cystatin C - a filtration marker insensitive to muscle mass - was measured in TEMPO3:4 but never reported, and call for reanalysis of those data. They further speculate that intermittent hypertonic dehydration events may actually worsen kidney injury, and highlight the psychological burden on patients who decline or discontinue tolvaptan due to its side effects. **Major Comments** **Comment 1:** *“1. Speculative muscle-mass loss and eGFR confounding The central premise - that tolvaptan induces sufficient muscle wasting to alter creatinine pools and confound eGFRcr - is plausible but unsupported by direct longitudinal body-composition or nutritional data from trial participants. The extrapolation of a 1.3% annual muscle-mass decline derives from general sarcopenia literature rather than ADPKD cohorts on tolvaptan, and the TEMPO3:4 trial did not report serial lean-mass measurements or nutritional assessments to substantiate clinically meaningful sarcopenia.”* **Response to Comment 1:** We agree with the reviewer that the TEMPO3:4 trial did not include serial assessments of muscle mass, body composition, or nutritional status and therefore does not provide direct evidence to substantiate clinically meaningful sarcopenia in this setting. Our hypothesis is therefore not intended to assert the presence of such effects, but to highlight a biologically plausible mechanism that could, in principle, influence creatinine-based eGFR (eGFRcr). To address this important limitation and to strengthen the empirical basis of our argument, we have substantially revised the manuscript by adding a dedicated section entitled “Detection of a clinically relevant drug effect on creatinine generation in clinical trials”. In this section, we move beyond conceptual reasoning and explicitly examine whether treatment-induced changes in endogenous creatinine generation of a magnitude sufficient to affect eGFRcr could be empirically detected or excluded within feasible study designs. Specifically, we:

quantify the magnitude of change in creatinine generation required to meaningfully influence eGFRcr trajectories based on calculations that use representative data from TEMPO3:4;
evaluate the detectability of such changes using direct and indirect measurement approaches (including imaging-based techniques, isotope dilution, and 24-hour urinary creatinine excretion), and;
(iii)    highlight that a within-trial, longitudinal evaluation of discordance between trajectories of creatinine-based eGFR and cystatin C-based eGFR (using cystatin C data that were prespecified and collected in TEMPO3:4), provides a practical and sufficiently powered approach to detect or refute such effects without the need for additional data collection.

In this way, the revised manuscript clarifies that, although direct measurements of muscle mass are lacking, the hypothesis is testable, as it can be evaluated using existing trial data and contemporary analytical approaches. **Comment 2:** *“2. Absence of published cystatin C results Although cystatin C was prespecified as a pharmacodynamic endpoint in TEMPO3:4, it is not clarified whether these data exist in full, nor discussed why they were omitted.[1] The authors of the TEMPO3:4 trial could investigate and report whether cystatin C trajectories differed between tolvaptan and placebo arms, and if not, whether non-significance or logistical constraints explain the absence of publication.”* **Response to Comment 2:** We agree that clarification regarding the availability and analysis of cystatin C data from the TEMPO3:4 trial is highly relevant, particularly in light of its prespecification as a pharmacodynamic endpoint, as well as its potential to inform the interpretation of eGFRcr in the presence of treatment-related changes in creatinine generation. As noted in the revised manuscript, cystatin C measurements were indeed included in the trial protocol, indicating that such data were collected. However, to our knowledge, analyses of cystatin C trajectories or derived cystatin C-based eGFR (eGFRcys) outcomes have not been reported. The reasons for this are not described in the original publications, and we have therefore refrained from speculating on this point. In the revised manuscript, we now explicitly highlight that the availability of these data provides a unique opportunity to evaluate discordance between eGFRcr and eGFRcys trajectories within the trial. Such analyses would allow assessment of whether treatment effects differ by filtration marker and could help clarify the extent to which creatinine-based estimates may be influenced by non-GFR determinants. This point is now more explicitly emphasised in the revised manuscript. **Comment 3:** *“3. Existing cystatin C literature in ADPKD Several studies (e.g., Sans et al., 2017) have evaluated cystatin C-based eGFR in early ADPKD stages and found concordance with eGFRcr, suggesting limited creatinine bias in this setting.[2] The manuscript would be strengthened by situating the hypothesis within this existing body of work.”* **Response to Comment 3:** We thank the reviewer for highlighting this important evidence. We agree that studies such as Sans et al. (2017) demonstrate a moderate correlation between eGFRcr and eGFRcys in patients with ADPKD, reflecting their shared dependence on underlying glomerular filtration rate. In that study, a correlation coefficient of 0.557 was observed at preserved values of eGFRcr and eGFRcys, indicating that approximately one-third of the variance is shared between the two measures (0.557×0.557×100%≈31%). At the same time, this correlation is incomplete, with a substantial proportion of variability remaining unexplained, consistent with contributions from non-GFR determinants such as creatinine generation for eGFRcr. Rather than contradicting our hypothesis, this observation provides the statistical basis for detecting treatment-induced divergence between eGFRcr and eGFRcys trajectories over time. To address the reviewer’s comment, we have now explicitly incorporated this perspective into the revised manuscript. The added text reads as follows: “In patients with ADPKD, eGFRcr and eGFRcys are moderately correlated, sharing approximately 30% of their variance (Ref: Sans L et al., PLoS One 2017;12(3):e0174583), consistent with their common dependence on true GFR. The remaining variance attributable to non-GFR determinants of each marker, including creatinine generation for eGFRcr, provides the statistical basis for detecting treatment-induced divergence between the two markers over time.” **Comment 4.** *“4. Psychological burden claims need empirical backing The discussion of guilt and self-blame among patients who forgo or stop tolvaptan is an important humanistic angle but remains conjectural. There is currently no published qualitative or quantitative research exploring patient-reported emotional outcomes in this context. The authors should recommend or initiate studies (e.g., structured interviews, validated quality-of-life instruments) to capture these dimensions.”* **Response to Comment 4:** We thank the reviewer for this suggestion and agree that the discussion of potential psychological burden, including feelings of guilt of self-blame among patients who decline of discontinue tolvaptan, remains speculative in the absence of empirical evidence. To address this point, we have revised the manuscript to explicitly acknowledge this limitation and to recommend further research in this area. Specifically, we now suggest that future studies should incorporate qualitative and quantitative approaches, such as the ones suggested by this reviewer, to systematically evaluate patient-reported emotional outcomes related to treatment decisions. The added text in the revised manuscript reads as follows: “Considering treatment discontinuation, the possibility of guilt and self-blame among patients who refuse to start or discontinue using tolvaptan currently remains largely conjectural, as qualitative or quantitative evidence on patient-reported emotional outcomes in this context is lacking. We recommend performance of studies (e.g., structured interviews, validated quality-of-life instruments) capable of capturing these dimensions.” **Comment 5.** *“5. Hypertonicity-related nephropathy hypothesis While episodes of volume depletion or hypertonicity could theoretically exacerbate kidney injury, the frequency, severity, and clinical impact of such events in tolvaptan-treated ADPKD patients remain undefined. Citing indirect evidence from heat-stress nephropathy does not substitute for ADPKD-specific cohort data. Prospective monitoring of electrolytes, osmolality, and acute GFR changes during intercurrent illnesses would be necessary to substantiate this concern.”* **Response to Comment 5:** We thank the reviewer for this thoughtful comment. We agree that, while episodes of volume depletion or hypertonicity could theoretically contribute to kidney injury, the frequency, severity, and clinical relevance of such events in tolvaptan-treated patients with ADPKD remain insufficiently characterised. We also concur that indirect evidence from heat-stress nephropathy cannot substitute for ADPKD-specific cohort data. To address this point, we have revised the manuscript to explicitly acknowledge these limitations and to emphasise the need for prospective evaluation. Specifically, we now highlight that studies incorporating longitudinal monitoring of electrolytes, osmolality, and acute changes in kidney function during intercurrent illness would be required to substantiate this hypothesis. The added text in the revised manuscript reads as follows: “While episodes of volume depletion or hypertonicity could theoretically exacerbate kidney injury, the frequency, severity, and clinical impact of such events in tolvaptan-treated patients with ADPKD remain undefined. At present, only indirect evidence from heat-stress nephropathy is available (Groothof D et al., Kidney360;5(12):1917-1921; Johnson R & Sánchez-Lozada L, Nat Rev Nephrol 2013;9(10):560-561; Yang X et al., BMC Nephrol 2020;21(1):159), which does not substitute for ADPKD-specific cohort data. Prospective monitoring of electrolytes, osmolality, and acute GFR changes during intercurrent illnesses would be necessary to substantiate this concern. Nonetheless, even this theoretical possibility of accelerated kidney injury underscores the need for analytical approaches capable of distinguishing true treatment effects on GFR from artefacts introduced by changes in creatinine generation.” **Minor comments** **Comment 6.** *“Body weight vs. composition: The manuscript notes stable body weight during TEMPO3:4, and suggests that fat-mass increases could mask lean-mass loss; recommending DXA or bioimpedance analyses in future trials would be helpful.”* **Response to Comment 6:** We thank the reviewer for highlighting this important point. The conceptual framework underlying our manuscript explicitly recognises that stable body weight does not preclude meaningful changes in body composition, and that changes in fat mass may indeed mask concurrent changes in lean tissue. We further acknowledge that the original version of the manuscript did not explicitly address how such discrepancies could be assessed in practice. We concur that discussing modalities capable of detecting these changes adds important methodological clarity. To address this, we have expanded the manuscript with a dedicated section entitled “Approaches to evaluate potential confounding related to changes muscle mass and creatinine generation in clinical trials”. In this section, we go beyond the assessment of muscle mass alone and emphasise that it is ultimately endogenous creatinine generation, rather than muscle mass per se, that is most relevant for the interpretation of eGFRcr. Specifically, we distinguish between scenarios in which changes in muscle mass and fat mass occur concordantly and scenarios in which these components change discordantly, for example due to alterations in muscle composition or fat redistribution. In the former cases, standard techniques such as dual-energy X-ray absorptiometry (DXA) or bioelectrical impedance analysis (BIA) can provide informative estimates, whereas in the case of discordant changes more advanced approaches capable of assessing total body creatine pools are required. These additions aim to clarify how potential discrepancies between body weight, body composition, and creatinine generation can be appropriately evaluated in future clinical and observational studies. **Comment 7:** *“Statistical framework: Propose power calculations and minimal detectable difference for a cystatin C - based reanalysis to guide feasibility.”* **Response to Comment 7:** We thank the reviewer for this helpful suggestion. In response to this comment, and in alignment with **Comment 1** of this reviewer, we have added a dedicated section entitled “Detection of a clinically relevant drug effect on creatinine generation in clinical trials”. As detailed in our **Response to Comment 1**, this section explicitly addresses the feasibility of a cystatin C-based re-analysis by providing quantitative estimates of the magnitude of treatment-induced changes in creatinine generation required to meaningfully influence eGFRcr, along with corresponding minimal detectable differences and sample size requirements under realistic assumptions. We further outline how a within-trial, longitudinal comparison of eGFRcr and eGFRcys trajectories in TEMPO3:4 can be used to detect such effects with adequate statistical power. We believe these additions directly address the reviewer’s request by demonstrating that a cystatin C-based re-analysis is not only conceptually appropriate, but also practically feasible within the existing trial dataset of TEMPO3:4. **Comment 8:** *“Figure clarity: In Figure 1, consider annotating with estimated effect sizes (e.g., expected decline in creatinine per kg muscle lost) to aid interpretability.”* **Response to Comment 8:** We thank the reviewer for this suggestion and agree that providing quantitative context is important for interpreting the plausibility of the proposed mechanism. Rather than incorporating numerical estimates directly in **Figure 1**, which is intended as a conceptual schematic illustration, we have chosen to present these calculations in a dedicated Box ( **Box 1**) in the manuscript. In this Box, we explicitly quantify the magnitude of change in endogenous creatinine generation (and corresponding muscle mass) that would be required to account for the observed difference in eGFRcr between treatment arms in the TEMPO3:4 trial, under clearly stated assumptions. In addition, the detectability and sample size implications of such changes are further elaborated in the section “Detection of a clinically relevant drug effect on creatinine generation in clinical trials” (see also our **Responses to Comments 1 and 7**). We believe that this approach maintains the clarity of Figure 1 while providing a transparent and appropriately contextualised quantitative framework elsewhere in the manuscript.

# Reviewer report: Reviewer response for version 1

**DOI:** 10.21956/openreseurope.22106.r55137

**Published:** 2025-07-09

**Authors:** Amaryllis van Craenenbroeck

**Affiliations:** 1 KU Leuven, Louvain, Belgium

I have read with great interest the letter by Groothof and Bakker questioning the kidney-protective effect of tolvaptan. Some comments: 

 - I am not sure whether the title fully covers the message (especially the adjective ‘biologically’). The authors hypothesize that use of tolvaptan can induce loss of muscle mass. This is an interesting, novel and challenging viewpoint and merits further investigation.

 However, in their further explanation, no discussion is present yet on including direct measurements of muscle mass/strength in future trials. In contrast with cystatin C based eGFR estimations, this would provide direct info on the ‘biological’ ground of the hypothesis. The same would be true for markers of malnutrition (part of the key hypothesis in the figure). I would suggest broadening this perspective a bit more.

 - A discussion on the effect of tolvaptan on change in kidney volume, the primary endpoint of the TEMPO3:4 trial, is lacking. The authors might want to discuss why their hypothesis would be more plausible than the underlying hypothesis of the TEMPO3:4 trial in a separate paragraph.

Where applicable, are recommendations and next steps explained clearly for others to follow? (Please consider whether others in the research community would be able to implement guidelines or recommendations and/or constructively engage in the debate)

Yes

Does the article adequately reference differing views and opinions?

Yes

Are all factual statements correct, and are statements and arguments made adequately supported by citations?

Yes

Is the rationale for the Open Letter provided in sufficient detail? (Please consider whether existing challenges in the field are outlined clearly and whether the purpose of the letter is explained)

Yes

Is the Open Letter written in accessible language? (Please consider whether all subject-specific terms, concepts and abbreviations are explained)

Yes

Reviewer Expertise:

nephrology, vascular dysfunction and its reversibility by exercise training

I confirm that I have read this submission and believe that I have an appropriate level of expertise to confirm that it is of an acceptable scientific standard, however I have significant reservations, as outlined above.

# Response

**Published:** 2026-05-17

**Authors:** Dion Groothof

**Affiliations:** Internal Medicine, University Medical Center Groningen, Groningen, Groningen, The Netherlands

**Detailed, itemised response to the comments by Reviewer Dr. Amaryllis van Craenenbroeck** I have read with great interest the letter by Groothof and Bakker questioning the kidney-protective effect of tolvaptan. Some comments: **Comment 1:** *“I am not sure whether the title fully covers the message (especially the adjective ‘biologically’). The authors hypothesize that use of tolvaptan can induce loss of muscle mass. This is an interesting, novel and challenging viewpoint and merits further investigation. However, in their further explanation, no discussion is present yet on including direct measurements of muscle mass/strength in future trials. In contrast with cystatin C based eGFR estimations, this would provide direct info on the ‘biological’ ground of the hypothesis. The same would be true for markers of malnutrition (part of the key hypothesis in the figure). I would suggest broadening this perspective a bit more.”* **Response to Comment 1:** We thank the reviewer for the thoughtful and constructive comment. We agree that, in the original version of the manuscript, the adjective ‘biologically’ in the title was insufficiently substantiated and that a broader discussion of direct measurements of muscle mass, muscle composition, and muscle strength would strengthen the biological basis of our hypothesis. To address this, we have made three substantive revisions. First, we have modified the title by replacing the terms ‘biologically plausible’ with ‘testable’, to more accurately reflect the nature of the hypothesis. Second, we have substantially expanded the manuscript to explicitly discuss how direct measurements of muscle mass, muscle composition, and muscle strength could be incorporated in future clinical trials, thereby broadening the biological perspective as suggested. Third, and importantly, we have added a new section demonstrating that treatment-induced changes in endogenous creatinine generation of clinically relevant magnitude are not only biologically plausible but also empirically detectable/testable using feasible study designs. In this context, we outline both direct approaches (such as imaging- and isotope-based quantification of creatine pools) and indirect approaches based on longitudinal discordance between trajectories of creatinine-based eGFR and cystatin C-based eGFR within existing trial datasets. We further emphasise in the concluding section how these measurement strategies can be employed into re-analyses of existing trials and integrated into future trial designs. **Comment 2:** “A discussion on the effect of tolvaptan on change in kidney volume, the primary endpoint of the TEMPO3:4 trial, is lacking. The authors might want to discuss why their hypothesis would be more plausible than the underlying hypothesis of the TEMPO3:4 trial in a separate paragraph.” **Response to Comment 2:** We thank the reviewer for this important comment and agree that the effects of tolvaptan on total kidney volume (TKV), the primary endpoint of the TEMPO3:4 trial, warrant explicit consideration in the context of our hypothesis. To address this, we have added a dedicated paragraph to the revised manuscript in which we discuss the observed temporal pattern of TKV growth rate reduction and its interpretation in the literature. In particular, we now acknowledge that early reductions in TKV growth may reflect a biologically plausible disease-modifying effect of tolvaptan, as previously emphasised. At the same time, we clarify that this biological interpretation does not preclude the possibility that concurrent mechanisms unrelated to GFR, as well as selective treatment discontinuation, may influence estimates of treatment effect on creatinine-based eGFR. Accordingly, we have revised the manuscript to explicitly position our hypothesis as complementary to, rather than in competition with, the established biological framework derived from TKV outcomes. The relevant text can be found in the revised manuscript in the section running from “A similar selection process may also help to explain…” to “…by highlighting potential sources of bias in the quantification of treatment effects on kidney function”.

# Reviewer report: Reviewer response for version 1

**DOI:** 10.21956/openreseurope.22106.r55601

**Published:** 2025-06-26

**Authors:** Michiel Betjes

**Affiliations:** 1 Erasmus Medical Center, Rotterdam, The Netherlands

The open letter bij Groothof and Bakker questions the kidney protective effect of tolvaptan in patients with polycystic disease. There main argument is that tolvaptan could potentially lower muscle mass thereby lowering the serum creatinine leading to positive effect on eGFR. This potential bias could be assessed by an alternative measurement of eGFR assessment by using cystatine C. 

 There are no data to support this hypothesis as cystatine C measurement and measurements on muscle mass from tolvaptan trials are not available/done. 

 The letter has value as a hypothesis is generated that questions the true effect of tolvaptan. The authors think this is an important issue as it is a matter of truth seeking and tolvaptan may be poorly tolerated. 

 Concerning the latter, the addition of a thiazide may reduce urinary output during tolvaptan while maintaining efficacy. This should be mentioned.

 Also, as stated by the authors, tolvaptan does reduce the growth of kidney cysts which is one of the most important predictors of the rate of eGFR loss. Thus, a biological plausible explanation for the action of tolvaptan is documented. Therefore, the sentence "We hypothesize that a reevaluation of the TEMPO3:4 trial data using the available cystatin C measurements would reveal no significant effect of tolvaptan on slowing kidney function decline compared with placebo" is to bold and should be rephrased to ".. could reveal a lesser efficacy of tolvaptan than based on creatine measurements..". 

 The need for cystatin C measurement if muscle mass loss could be expected is in itself an important message. Given the side effects of tolvaptan, balancing these against the true efficacy in slowing down eGFR loss is another important message.

 In conclusion, the hypothesis has no measured data to build on but in itself is of interest as it highlights that serum creatine may sometimes be a doggy measure of establishing kidney function. I would recommend to rewrite the letter such that this is the main message and take tolvaptan trials as an example of study design that would have benefited from addition of cystatin measurement. In addition, this could still be done in the recently started HYDROPROTECT trial.

Where applicable, are recommendations and next steps explained clearly for others to follow? (Please consider whether others in the research community would be able to implement guidelines or recommendations and/or constructively engage in the debate)

Yes

Does the article adequately reference differing views and opinions?

Partly

Are all factual statements correct, and are statements and arguments made adequately supported by citations?

Yes

Is the rationale for the Open Letter provided in sufficient detail? (Please consider whether existing challenges in the field are outlined clearly and whether the purpose of the letter is explained)

Yes

Is the Open Letter written in accessible language? (Please consider whether all subject-specific terms, concepts and abbreviations are explained)

Yes

Reviewer Expertise:

general nephrology and kidney transplantation

I confirm that I have read this submission and believe that I have an appropriate level of expertise to confirm that it is of an acceptable scientific standard, however I have significant reservations, as outlined above.

# Response

**Published:** 2026-05-17

**Authors:** Dion Groothof

**Affiliations:** Internal Medicine, University Medical Center Groningen, Groningen, Groningen, The Netherlands

**Detailed, itemised response to the comments of Reviewer Dr. Michiel Betjes**   The open letter bij Groothof and Bakker questions the kidney protective effect of tolvaptan in patients with polycystic disease. There main argument is that tolvaptan could potentially lower muscle mass thereby lowering the serum creatinine leading to positive effect on eGFR. This potential bias could be assessed by an alternative measurement of eGFR assessment by using cystatine C. **Comment 1:** *“There are no data to support this hypothesis as cystatine C measurement and measurements on muscle mass from tolvaptan trials are not available/done.”* **Response to Comment 1:** We thank the Reviewer for this important comment. We agree that there are no direct data on muscle mass from the pivotal tolvaptan trials to support our hypothesis. However, we would like to clarify that cystatin C measurements are in fact available in the pivotal TEMPO3:4 trial (Torres VE et al., N Engl J Med 2012;367(25):2407-2418). As specified in the trial protocol, published as Supplementary Material of the original report, cystatin C was included as a prespecified pharmacodynamic variable, alongside repeated measurement of creatinine as a secondary efficacy endpoint. The availability of these longitudinal data enables, in principle, a within-trial, trajectory-based assessment of divergence between creatinine-based eGFR (eGFRcr) and cystatin C-based eGFR (eGFRcys). As outlined in the revised manuscript, such an approach would allow detection of small but clinically relevant treatment-induced changes in endogenous creatinine generation with adequate statistical power, leveraging the existing sample size and repeated measurements collected during the trial. We have clarified and expanded this point in the revised manuscript, which can be found in between lines “Although evaluation of discordance between eGFRcr and eGFRcys trajectories requires substantially larger sample sizes than approaches based on 24-hour urinary creatinine excretion under ideal conditions, it offers a unique practical advantage” through “…, this approach offers substantially greater statistical power and practical feasibility than conducting a separate short-term study with two repeated measurements.” We believe that this addition addresses the Reviewer’s concern by demonstrating that, while direct measurements of muscle mass are indeed lacking, the available data nonetheless permit an indirect but empirically testable evaluation of the proposed hypothesis. **Comment 2:** *“The letter has value as a hypothesis is generated that questions the true effect of tolvaptan. The authors think this is an important issue as it is a matter of truth seeking and tolvaptan may be poorly tolerated.”* **Response to Comment 2:** We thank the reviewer for these supportive comments and for recognising the relevance of the hypothesis presented. **Comment 3:** *“Concerning the latter, the addition of a thiazide may reduce urinary output during tolvaptan while maintaining efficacy. This should be mentioned.”* **Response to Comment 3:** We thank the Reviewer for this valuable suggestion and agree that the potential for thiazide diuretics to mitigate aquaretic side effects of tolvaptan is clinically relevant and warrants mention. Accordingly, we have revised the manuscript to include discussion of this approach in the context of the ongoing HYDRO-PROTECT trial, which evaluates whether hydrochlorothiazide can be added to tolvaptan to reduce urinary output without compromising efficacy. As noted in the revised text, this strategy directly addresses a key determinant of and treatment discontinuation. At the same time, we clarify that whether efficacy is fully preserved remains under investigation, and we highlight that the current trial design does not include cystatin C measurements, which limits the ability to assess potential effects on creatinine generation using the approaches discussed in our manuscript. **Comment 4:** *“Also, as stated by the authors, tolvaptan does reduce the growth of kidney cysts which is one of the most important predictors of the rate of eGFR loss. Thus, a biological plausible explanation for the action of tolvaptan is documented. Therefore, the sentence "We hypothesize that a reevaluation of the TEMPO3:4 trial data using the available cystatin C measurements would reveal no significant effect of tolvaptan on slowing kidney function decline compared with placebo" is to bold and should be rephrased to ".. could reveal a lesser efficacy of tolvaptan than based on creatine measurements..".”* **Response to Comment 4:** We agree that tolvaptan has a biologically plausible mechanism of action, as reflected by its demonstrated effect on reducing the growth of kidney cysts, a key predictor of subsequent decline in eGFR. We also agree that our original statement was too strong in light of this evidence. To address this, we have removed the statement from the abstract and have used more nuanced stance in the Introduction to adopt a more balanced interpretation, placing emphasis on the uncertainty regarding both the direction and true magnitude of tolvaptan’s effect on GFR preservation. In addition, this comment, together with aspects of other Reviewers’ comments, prompted us to further expand the manuscript with a dedicated section on “Missing data, informative dropout, and model validity”. In this section, we outline how alternative analytical approaches could either attenuate or strengthen the estimated treatment effect, depending on the underlying assumptions regarding missing data. This revision clarifies that our objective is not to refute the biological plausibility of tolvaptan’s effect, but to highlight the importance of fully evaluating potential sources of bias in its quantification. **Comment 5:** *“The need for cystatin C measurement if muscle mass loss could be expected is in itself an important message. Given the side effects of tolvaptan, balancing these against the true efficacy in slowing down eGFR loss is another important message.”* **Response to Comment 5:** We thank the Reviewer for highlighting these important points and agree that the need for cystatin C measurement in settings where changes in muscle mass or creatinine generation could be expected is a key message of our work. We also agree that, given the side-effect profile of tolvaptan, careful balancing of tolerability against true efficacy in slowing eGFR decline is clinically highly relevant. To address this, we revised the manuscript to place greater emphasis on the role of cystatin C as a complementary filtration marker. In particular, we expanded the discussion to more clearly describe how combined analyses of eGFRcr and eGFRcys can be used to detect or exclude treatment-related changes in muscle mass and creatinine generation, thereby improving interpretation of treatment effects. **Comment 6:** *“In conclusion, the hypothesis has no measured data to build on but in itself is of interest as it highlights that serum creatine may sometimes be a doggy measure of establishing kidney function. I would recommend to rewrite the letter such that this is the main message and take tolvaptan trials as an example of study design that would have benefited from addition of cystatin measurement. In addition, this could still be done in the recently started HYDROPROTECT trial.”* **Response to Comment 6:** We thank the Reviewer for this thoughtful recommendation. In line with this suggestion, we have revised the manuscript to more clearly position the central message that reliance on creatinine alone may be insufficient to fully characterise treatment effects on kidney function, particularly in settings where non-GFR determinants such as creatinine generation may be altered. We now present the tolvaptan trials as a representative example of a study context in which complementary assessment of cystatin C could have strengthened interpretation of treatment effects. We also refer to our **Response to Comment 5** of this reviewer. In addition, we have incorporated discussion of the ongoing HYDRO-PROTECT trial, noting that inclusion of cystatin C measurements in such studies would further enhance the ability to evaluate potential effects on creatinine generation and improve robustness of outcome assessment.
